# Supplementary material for: The Intransitive Logic of Directed Cycles and Flipons Enhances the Evolution of Molecular Computers by Augmenting the Kolmogorov Complexity of Genomes
Source: Int J Mol Sci. 2023 Nov 18;24(22):16482. doi: 10.3390/ijms242216482 (PMC10671625; doi:10.3390/ijms242216482)
Supplement: Supplementary file 1 [file ijms-24-16482-s001.zip › ijms-2644033-supplementary.pdf]

Supplemental Figure

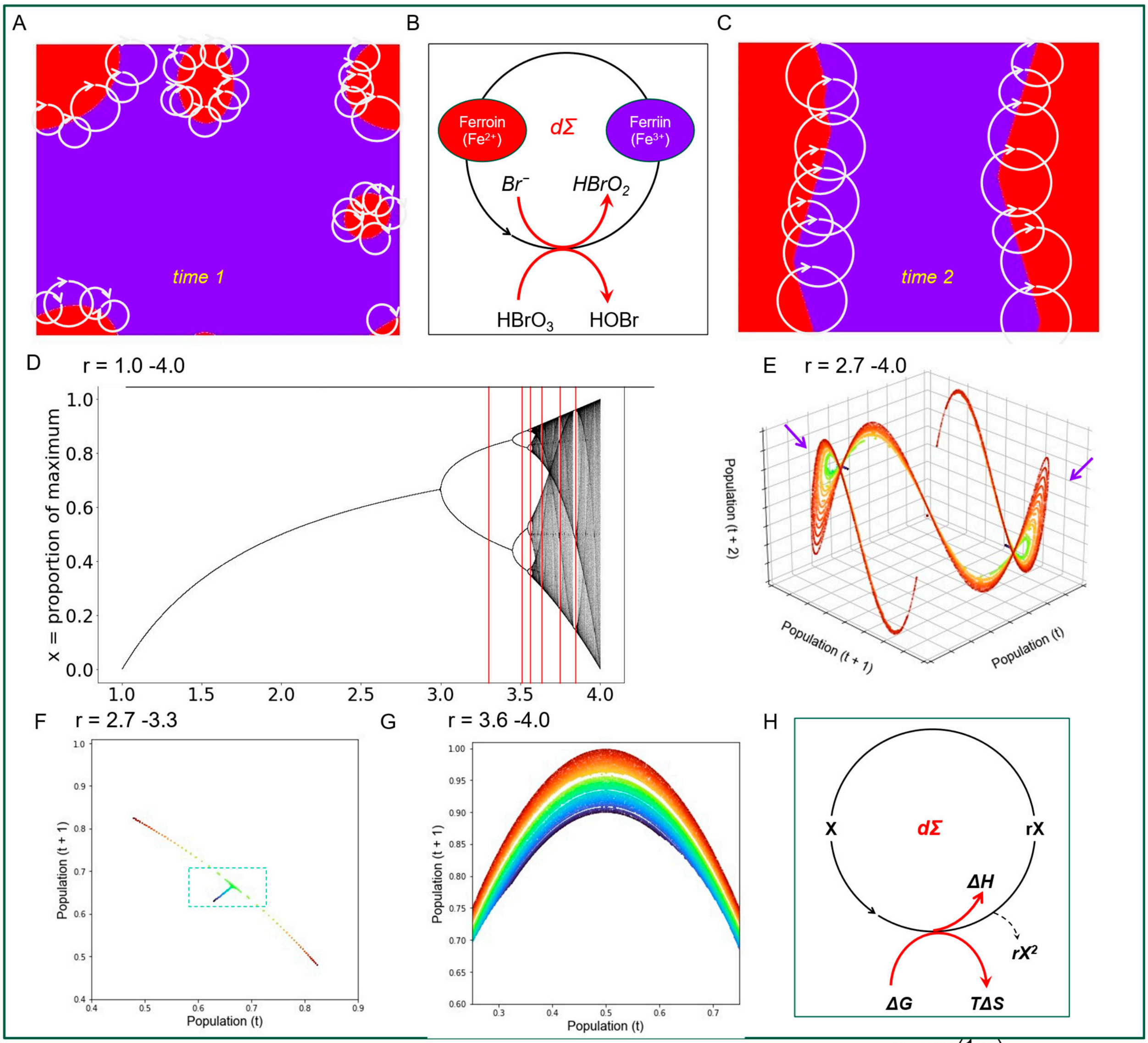

$x_{t+1}=rx_t(1-x_t)$

Supplemental Figure S1. **Different Examples of Dissipative Structures**. A. Computer modeling of the Belousov-Zhabotinsky reaction shown in the center panel. The red and blue color changes correspond to the oxidation states of iron (Fe). The boundaries mark the region of transition between the two phases and are very sharp. The Be-Zh cycles that define the boundaries are drawn in white. B. The conversions that drive the reaction are given with the red arrows. The reduction of iron from blue to red is driven by the spontaneous formation of Br_2 in the solution. C**.** A later time point D. Population recurrence map for the logistic equation $x_{t+1} = rx_t(1-x_t)$, s where x_t is the proportion of the maximum possible population at time *t* and *r* allows for different rates of population increase as the cycle reiterates. E. The cycles for r = 3.2 for 3,000 cycles measure at times *t*, *t+1* an *t*+2. F. Recurrence map for r = 2.7 -3.3 for *t* and *t+1*. The dotted box highlights the region where a DC forms when the Lyapunov $\lambda \approx 0$. G**.** Recurrence map for r =3.6 -4.0 for *t* and *t+1* showing the non-overlapping paths generated as the cycle reiterates**.** H. At each step, the population grows by *rx* and decreases by rx^2, where *r* is the rate of increase, where *x* is the ratio of the current population to the maximal possible number.
